# Clinical Outcomes of Medial Meniscal Allograft Transplantation With or Without High Tibial Osteotomy: A Case-Control Study Up to 8 Years of Follow-up

**DOI:** 10.1177/03635465241248822

**Published:** 2024-05-20

**Authors:** Alberto Grassi, Gian Andrea Lucidi, Stefano Di Paolo, Emanuele Altovino, Piero Agostinone, Giacomo Dal Fabbro, Iacopo Romandini, Giuseppe Filardo, Stefano Zaffagnini

**Affiliations:** * Clinica Ortopedica e Traumatologica II, IRCCS Istituto Ortopedico Rizzoli, Bologna, Italy; ‡ Surgical Department, Aspetar Hospital, Qatar, Doha; § Applied and Translational Research Center, IRCCS Istituto Ortopedico Rizzoli, Bologna, Italy; ∥ Faculty of Biomedical Sciences, Università della Svizzera Italiana, Lugano, Switzerland; ¶ Dipartimento di Scienze Biomediche e Neuromotorie DIBINEM, Università di Bologna, Bologna, Italy; Investigation performed at Istituto Ortopedico Rizzoli di Bologna, Bologna, Italy

**Keywords:** allograft, osteotomy, knee, meniscus

## Abstract

**Background:**

Satisfactory clinical results of meniscal allograft transplantation (MAT) have been reported in recent years. However, it remains unclear whether the clinical outcomes of MAT when combined with an osteotomy are inferior to those of isolated MAT.

**Purpose:**

To compare the survival rates and clinical outcomes of patients who received isolated medial MAT with those of patients undergoing medial MAT combined with high tibial osteotomy (HTO).

**Study Design:**

Cohort study; Level of evidence, 3.

**Methods:**

A total of 55 patients underwent arthroscopic medial MAT using the soft tissue technique and HTO (mean age, 41.3 ± 10.4 years; 9 female); after fuzzy case-control matching on demographics, 55 controls who underwent isolated medial MAT were also included. Survival analyses were performed using the Kaplan-Meier method with surgical failure, clinical failure (Lysholm score, <65), and reoperation as endpoints. Subjective clinical scores were collected preoperatively and at the final follow-up.

**Results:**

The mean follow-up time was 5.4 years, up to 8 years. All outcomes significantly improved at the last follow-up (*P* < .001). No differences were identified between MAT and MAT + HTO groups preoperatively and at the last follow-up (*P* > .05). At the final follow-up, 8 of 55 (14.5%) of the MAT + HTO patients and 9 of 55 (16.4%) of the MAT patients had a Lysholm score <65 (*P* = .885). Overall, 90% of the patients declared they would repeat the surgery regardless of the combined procedure. Surgical failure was identified in 6 of 110 (5.5%) patients: 5 of 55 (9.1%) in the MAT + HTO group and 1 of 55 (1.8%) in the MAT group (*P* = .093). Clinical failure was identified in 19 of 110 (17.3%) patients: 11 of 55 (20%) in the MAT + HTO group and 8 of 55 (14.5%) in the MAT group (*P* = .447). A significantly lower survivorship from surgical failure was identified in the MAT + HTO group (hazard ratio, 5.1; *P* = .049), while no differences in survivorship from reoperation and clinical failure were identified (*P* > .05).

**Conclusion:**

Patients undergoing medial MAT + HTO showed similar clinical results to patients undergoing isolated medial MAT at midterm follow-up, and thus a surgically addressed malalignment does not represent a contraindication for medial MAT. However, the need for a concomitant HTO is associated with a slightly higher failure rate over time.

Meniscal allograft transplantation (MAT) is a valid treatment for postmeniscectomy syndrome, referred to as a symptomatic unicompartmental pain in the meniscus-deficient knee.^4^ The goal of MAT is to limit the negative effects of meniscal loss, which means relief of pain, restoration of joint biomechanics, improvement in knee function, and prevention of osteoarthritis.^6^ The ideal candidate for MAT is a young patient, usually ≤50 years of age, with a history of total or subtotal meniscectomy in an otherwise stable and aligned knee, having pain at the corresponding joint compartment, and not responding to nonoperative treatment.^4^ Uncorrected axial malalignment and the presence of concomitant knee pathologies are considered at least relative, if not absolute, contraindications for meniscal replacement surgeries.^4^ Regarding limb alignment, MAT combined with a realignment procedure such as high tibial osteotomy (HTO) is a controversial topic in the literature.

The International Meniscus Reconstruction Experts Forum consensus statement recommends that a realignment osteotomy should be considered in the presence of a mechanical axis deviation.^6^ Although satisfactory clinical results of MAT have been reported, most studies did not differentiate between the effects of isolated MAT and those of MAT combined with realignment procedures. It therefore remains unclear whether the clinical outcomes of MAT + HTO are inferior to those of isolated MAT. Furthermore, matched groups that included MAT with and without an HTO have not yet been studied. The purpose of this study was to compare the survival rates and clinical outcomes of patients who received isolated medial MAT with those of patients who underwent medial MAT + HTO, to define if a surgically corrected varus malignment may affect patients’ outcomes and the survivorship of MAT over time. We hypothesized that there was no difference between the 2 groups.

## Methods

### Patient Selection and Matching

All the MATs performed at a single institution (Rizzoli Orthopaedic Institute) between June 2004 and April 2019 were screened and assessed for eligibility. The indications for MAT were unicompartmental pain due to a previous total or subtotal meniscectomy, with osteoarthritis grade 1 to 3 according to the Kellgren-Lawrence radiographic evaluation; no signs of contralateral compartment or patellofemoral compartment damage; and <5° of coronal malalignment. In patients who had >5° of varus malalignment, a corrective HTO was performed. All patients undergoing MAT were adequately counseled regarding the risks and benefits of the procedure as well as surgical alternatives.^9^

The inclusion criteria for the present study were patients who had undergone isolated medial MAT and medial MAT + HTO who had reached the minimum follow-up of 2 years. No other exclusion criteria were applied.

**Figure 1.**
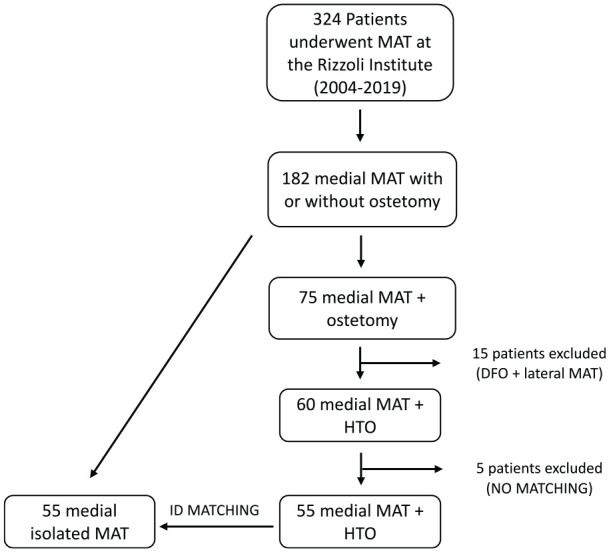
Patient selection flowchart. Case-control matching was performed for sex, age at surgery, body mass index at surgery, presence of previous cartilage procedures, presence of previous anterior cruciate ligament surgery, and time to return to sport. DFO, distal femoral osteotomy; HTO, high tibial osteotomy; MAT, meniscal allograft transplantation.

From the overall cohort of 324 patients, only the patients undergoing a medial MAT with or without HTO were included (182 patients). From this cohort, patients undergoing MAT + HTO procedures were considered cases and assigned to the MAT + HTO group (Figure 1).

Case-control matching was performed in SPSS (Version 26; IBM Corp). Matched controls undergoing only MAT surgery were assigned to the MAT group. Case-control matching was performed for sex (binary; male or female), age at surgery (continuous; tolerance 2 years), body mass index at surgery (continuous; tolerance 2 points), presence of previous cartilage procedures (binary; yes or no), presence of previous anterior cruciate ligament surgery (binary; yes or no), and time to return to sport (continuous; tolerance 2 months).

### Surgical Technique

For MAT, fresh-frozen (−80°C) nonirradiated and non–antigen matched allografts were used. The transplantation was performed arthroscopically using a double-tunnel^7^ technique and a meniscal allograft without bone plugs. Peripheral suture to the capsule was performed with all-inside stitches (nonabsorbable ULTRABRAID No. 0 wire and poly-L-lactide bio-absorbable implants, Smith & Nephew; TRUESPAN, DePuy Synthes, Johnson & Johnson). Anterior and posterior horns were fixed with a transosseous suture. After checking for graft stability, in case of a varus deformity of ≥5°, we performed a concomitant lateral closing-wedge HTO. The planned alignment correction was aimed to reach a postoperative value of hip-knee-ankle angle as near as possible to a neutral mechanical alignment (hip-knee-ankle angle, 180°-182°), preserving as much as possible the joint line obliquity from excessive change, considered as a postoperative mechanical medial proximal tibial angle >95°. The mechanical realignment was verified using fluoroscopy, and the fixation was performed with a Krakow staple. Further details on meniscal sizing, surgical steps of MAT + HTO, and rehabilitation protocols are provided in previous studies.^8,14^ In particular, the rehabilitation protocol did not vary between the 2 groups, and it started with 2 weeks of immobilization and no weightbearing, followed by toe-touch weightbearing for the following 2 weeks, restriction of range of motion (0°-90° during weeks 3-4 and then free range of motion), isometric exercises, and closed kinetic chain strengthening. At week 4 postoperatively, partial weightbearing was allowed, and at week 6 postoperatively, progression to full weightbearing was started; full flexion of the knee was also allowed.

### Patient Evaluation

Demographic details such as age, sex, limb, age at surgery, height, and weight were extracted from medical records. Furthermore, the years from the first meniscectomy, the cartilage status, and the details of surgical intervention and concomitant procedures were obtained. All patients were contacted by telephone by 2 investigators (I.R., E.A.) and underwent an interview with questionnaires.^15^ The preoperative evaluation was performed with the Lysholm score, Tegner Activity Scale score, Knee injury and Osteoarthritis Outcome Score, and a visual analog scale score based on 0 to 100.^3^ The same evaluations were repeated at 2 years, 5 years, and the final follow-up, which reached 8 years. Moreover, all the surgical procedures performed during the follow-up period were recorded.

Based on these data, 2 different criteria for failure were used. Surgical failure was considered in the case of revision procedures related to the initial MAT, such as graft tear or revision of MAT, unicompartmental knee arthroplasty, and total knee arthroplasty. In the case of surgical failure, clinical scores were not administered at the time of the interview, and the time from MAT to revision surgery was noted and used for survival analysis. Clinical failure was considered in the case of the revision procedures listed previously and also in the case of a poor Lysholm score (<65 points) at the final follow-up.^3^ In the case of clinical failure, the survival time was referred to as the time when the follow-up was collected, and therefore this temporal landmark was considered the time to failure in the survival analysis.^8^

### Ethics

The present study was approved by the local ethics committee of the Rizzoli Orthopaedic Institute, Bologna, Italy (prot. gen. n. 0026385). All patients undergoing isolated MAT or MAT + HTO were adequately counseled regarding the risks and benefits of the procedures and surgical alternatives, and provided signed informed consent forms.

### Statistical Analysis

Continuous parametric variables are expressed as mean ± SD with 95% CI, noncontinuous variables are presented as median and interquartile range, and categorical variables are expressed as number and percentage. The paired Student *t* test and Wilcoxon signed-rank test were used to compare MAT and MAT + HTO groups for continuous and noncontinuous variables, respectively. The chi-square test was used to compare frequencies between groups. Bonferroni correction for the *P* values was used to account for the group and time effect.

Survival analyses were performed using the Kaplan-Meier method. The patients obtaining a Lysholm score <65 at the follow-up interview were considered to have experienced clinical failure. Surgical failure was determined as revision procedures related to the initial MAT, such as graft tear or revision of MAT, unicompartmental knee arthroplasty, and total knee arthroplasty. When present, the surgical failure time was retrieved to not overestimate the survival time. Reoperation was determined as any other procedures that did not involve the meniscus being transplanted, such as plate removal, ligament reconstruction, arthrolysis, or cartilage procedures. Surgical failure, surgical or clinical failure, and reoperations were used as separate endpoints for the survival analysis. Survival proportions at 2 years, 5 years, and the final follow-up with standard errors were calculated. Hazard ratios with 95% CIs were also reported. Differences were considered significant with *P* < .05. All the statistical analyses were performed in SPSS.

An a priori power analysis was performed in G*Power (Version 3.1) based on the results of a previous similar study that analyzed the failure rate of MAT.^17^ At 5 years of follow-up, a difference of 16% in survival rate was reported between isolated medial MAT and medial MAT + HTO.^17^ Adopting a Fisher exact test to compare survival rates between the 2 groups, we determined that ≥98 participants (49 per group) were required to have a power of 0.9 and a type I error of .05.

## Results

A total of 110 patients—55 MAT + HTO cases and 55 matched MAT controls—were included in the analysis. The mean follow-up time of both groups was 5.4 years: 4.6 ± 3.4 years [95% CI, 3.8-5.5 years] for MAT and 6.1 ± 3.2 years [95% CI, 5.2-7.0 years] for MAT + HTO. Overall, 32 patients, 16 (29%) from each group, had a previous anterior cruciate ligament surgery and 9 patients, 5 (9%) in the MAT group and 4 (7%) in the MAT + HTO group, had a previous cartilage procedure (Table 1). No differences in demographics emerged between the 2 groups except for follow-up time, which was longer in the MAT + HTO group (*P* = .032). Time to return to sport was 8.7 ± 4.7 months [95% CI, 6.9-10.4 months] for MAT and 8.6 ± 5.1 months [95% CI, 6.8-10.4 months] for MAT + HTO (*P* = .333).

**Table 1:** Patient Characteristics^*a*^

	MAT	MAT + HTO	*P*
Sex, male/female, n	46/9	46/9	>.999
Limb, right/left, n	30/25	35/20	.438
Age at surgery, y	42.9 ± 8.7 [40.6-45.2]	41.3 ± 10.4 [38.6-44.1]	.067
Height, cm	176 ± 7.9 [173.9-178.1]	176.2 ± 8.6 [174-178.5]	.954
Weight, kg	79.1 ± 12.1 [75.9-82.3]	79.7 ± 14.6 [75.9-83.6]	.743
BMI	25.2 ± 2.9 [24.4-26.1]	25.5 ± 3.6 [24.6-26.5]	.670
Previous ACL surgery, n (%)	16 (29)	16 (29)	>.999
Primary/revision, n	9/7	8/8	>.999
Previous cartilage procedures, n (%)	5 (9)	4 (7)	>.999
Follow-up time, y	4.6 ± 3.4 [3.8-5.5]	6.1 ± 3.2 [5.2-7.0]	.032
Time to return to sport, mo	8.7 ± 4.7 [6.9-10.4]	8.6 ± 5.1 [6.8-10.4]	.333

a Data are presented as mean ± SD [95% CI] unless otherwise indicated. ACL, anterior cruciate ligament; BMI, body mass index; HTO, high tibial osteotomy; MAT, meniscal allograft transplantation.

### Clinical Scores

All outcomes significantly improved at the last follow-up (*P* < .001). Overall, improvements of 36.2, 30.6, 1.6, and 40.4 points were found for the visual analog scale score, Lysholm score, Tegner score, and Knee injury and Osteoarthritis Outcome Score, respectively. No differences were identified between MAT and MAT + HTO groups preoperatively and at the last follow-ups (*P* > .05). At the final follow-up, 8 of 55 (14.5%) of the MAT + HTO patients and 9 of 55 (16.4%) of the MAT patients had a Lysholm score <65 (*P* = .885). Overall, 90% of the patients declared they would repeat the surgery regardless of the combined procedure (Table 2).

**Table 2:** Clinical Outcomes Preoperatively and at the Last Follow-up^*a*^

	Preoperative		Last Follow-up		*P*: Time Effect		*P*: Group Effect	
	MAT	MAT + HTO	MAT	MAT + HTO	MAT (preoperative vs postoperative)	MAT + HTO (preoperative vs postoperative)	MAT vs MAT + HTO (preoperative)	MAT vs MAT + HTO (postoperative)
VAS score	71.0 ± 18.9	65.4 ± 24.0	29.0 ± 24.3	34.9 ± 29.2	<.001	<.001	.290	.220
Lysholm score	48.6 ± 17.7	54.2 ± 18.6	81.4 ± 17.2	83.1 ± 17.5	<.001	<.001	.135	.730
Lysholm score <65^*b*^	34/55 (62)	43/55 (78)	8/55 (15)	9/55 (16)	<.001	<.001	.068	.885
Tegner score	2.0 [1.1-3.2]	3.1 [1.4- 4.2]	4.2 [3.2- 5.3]	4.3 [3.1-5.5]	<.001	.009	.012	.825
KOOS								
Symptoms	39.1 ± 18.9	40.2 ± 22.8	79.1 ± 17.3	82.7 ± 16.8	<.001	<.001	.455	.186
Pain	49.5 ± 21.6	47.1 ± 23.4	85.0 ± 15.3	86.2 ± 17.5	<.001	<.001	.373	.680
Sport and Recreation	28.0 ± 22.9	27.5 ± 25.1	56.3 ± 29.9	61.6 ± 34.0	<.001	<.001	.868	.360
ADL	46.6 ± 25.9	46.1 ± 26.0	92.0 ± 11.2	91.4 ± 16.7	<.001	<.001	.483	.831
QoL	31.0 ± 17.4	30.7 ± 20.7	68.6 ± 28.5	69.8 ± 27.7	<.001	<.001	.901	.815
Self-score			72.7 ± 19.2	73.4 ± 21.4				.923
Satisfaction score			78.1 ± 20.7	76.2 ± 27.7				.637
Would repeat surgery^*b*^			50/55 (91)	49/55 (89)				.728

a Data are presented as mean ± SD [95% CI], median and interquartile range, or n (%). ADL, Activities of Daily Living; HTO, high tibial osteotomy; KOOS, Knee injury and Osteoarthritis Outcome Score; MAT, meniscal allograft transplantation; QoL, Quality of Life; VAS, visual analog scale.

b Chi-square test used instead of *t* test. Last follow-up is the last follow-up available for each patient (mean, 6.0 years).

### Reoperations and Failures

Reoperation was required in 23 of 110 (20.9%) patients. Of these, 14 of 55 (25%) occurred in the MAT + HTO group, while 9 of 55 (16.4%) occurred in the MAT group (*P* = .262). The full list of reoperation causes is presented in Table 3. Surgical failure was identified in 6 of 110 (5.5%) patients, with 5 of 55 (9.1%) occurring in the MAT + HTO group and 1 of 55 (1.8%) in the MAT group (*P* = .093). Clinical failure was identified in 19 of 110 (17.3%) patients, with 11 of 55 (20%) occurring in the MAT + HTO group and 8 of 55 (14.5%) in the MAT group (*P* = .447).

**Table 3:** Causes of Reoperation^*a*^

Surgery	MAT (n)	MAT + HTO (n)
Surgical failures		
Total knee arthroplasty	0	2
Allograft resection	1	3
Other reoperations		
Autologous chondral implant	1	0
Arthroscopic arthrolysis	2	2
Arthroscopic debridement	1	0
Stem cell injection	3	1
Hardware removal	0	2
HTO	1	1
Mobilization under anesthesia	0	3
Total	9	14

a HTO, high tibial osteotomy; MAT, meniscal allograft transplantation.

### Survival Analysis

**Figure 2.**
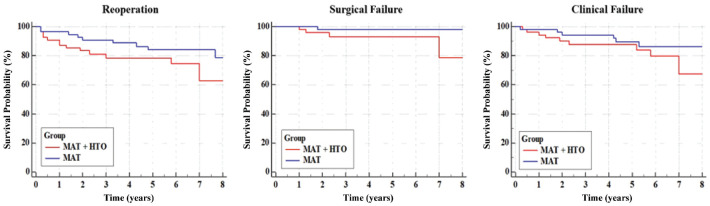
Kaplan-Meier curves describing the survivorship of the meniscal allograft transplantation (MAT) + high tibial osteotomy (HTO) (cases light grey) and MAT (controls dark grey) groups considering reoperations (left), only surgical failure (middle), and clinical failure (right). The presence of HTO significantly affected the survival rate from surgical failure (hazard ratio, 5.1; *P* = .049).

No differences in survivorship from reoperation were found between the MAT + HTO and MAT groups (hazard ratio [HR], 1.9 [95% CI, 0.8-4.3], *P* = .133). A significantly lower survivorship from surgical failure rate was identified in the MAT + HTO group (HR, 5.1 [95% CI, 1.0-26.0], *P* = .049), while no differences were found in clinical failure (HR, 2.2 [95% CI, 0.8-5.6], *P* = .109) (Table 4, Figure 2).

**Table 4:** Survival Rates^*a*^

	Events/Total Cases (%)	Survival Rate, % (SE)			*P*	HR [95% CI]
		2 y	5 y	8 y		
Reoperation						
Overall	23/110 (20.9)	87.2 (3.2)	81.2 (3.9)	71.8 (5.7)		
MAT	9/55 (16.3)	90.1 (3.9)	84.0 (5.3)	78.8 (7.1)		
MAT + HTO	14/55 (25.5)	83.6 (5.0)	78.5 (5.9)	63.1 (9.4)	.133	1.9 [0.8-4.3]
Surgical failure						
Overall	6/110 (5.5)	97.0 (1.7)	95.9 (2.0)	90.0 (4.4)		
MAT	1/55 (1.8)	98.1 (1.9)	98.1 (1.9)	98.1 (1.9)		
MAT + HTO	5/55 (9.1)	96.0 (2.8)	93.1 (3.9)	78.8 (9.9)	.049	5.1 [1.0-26.0]
Clinical failure						
Overall	19/110 (17.3)	92.3 (2.6)	88.5 (3.3)	78.3 (5.3)		
MAT	8/55 (14.5)	94.4 (3.1)	89.6 (4.5)	86.5 (5.3)		
MAT + HTO	11/55 (20.0)	90.3 (4.2)	87.6 (4.8)	67.5 (9.9)	.109	2.2 [0.8-5.6]

a HR, hazard ratio; HTO, high tibial osteotomy; MAT, meniscal allograft transplantation.

## Discussion

The most important finding of the present study is that medial MAT + HTO provided satisfactory survival from clinical and surgical failures at a midterm follow-up. The need for a concomitant HTO when performing MAT led to overall similar clinical outcomes compared with isolated MAT in aligned knees.

The results of the present study contribute to further defining the role and outcomes of HTO as an associated procedure to MAT in patients with combined medial meniscal deficiency and varus malalignment. Indeed, because of the multiple knee comorbidities frequently associated with patients requiring MAT, it is challenging to discern whether positive clinical outcomes are correlated with the replacement of the meniscus or associated surgeries such as HTO.^6^ Moreover, although the International Meniscus Reconstruction Experts Forum^6^ strongly recommends a realignment osteotomy in case of an unfavorable mechanical axis, there is still no consensus on several aspects, including the ideal surgical approach, the threshold of axial deviation to perform HTO, the amount of correction, and the expected results. The literature lacks conclusive evidence regarding the impact of associated HTO on the patient-reported outcome measures (PROMs) when performing MAT.

An evaluation of 22 patients who underwent medial MAT + HTO reported excellent clinical results and a low failure rate (5%) at 10 years of follow-up.^13^ In that study, all the patients returned to work, and 90% of them resumed sports activity at a mean 10 months after surgery. Similarly, a survivorship analysis of 39 medial MATs performed with an open procedure and viable allograft found an increased survival rate in patients requiring concomitant HTO.^17^ In this study, the survival rate increased from 74% to 83% at 10 years of follow-up in the presence of an osteotomy associated with MAT.^17^ Verdonk et al^18^ reported significantly higher PROMs and less swelling and pain in patients with medial MAT + HTO compared with patients requiring isolated MAT. However, there was no difference between the 2 groups in cartilage degeneration over time, graft extrusion, and signal intensity at the magnetic resonance imaging evaluation. On the other hand, a recent study on an active population found that realignment osteotomy was correlated with a doubled risk of short-term clinical failure, defined as persistent knee disability and pain after MAT.^22^

Several systematic reviews have analyzed prognostic factors of survivorship of meniscal allografts with or without concomitant procedures. A systematic review that included 24 studies reported no significant difference in postoperative patient-reported outcomes in isolated MAT versus combined MAT; however, no sufficient data were found on the effects of osteotomies on meniscal allograft.^12^ A study by Novaretti et al^15^ analyzed survivorship and outcomes of MAT with a minimum 10-year follow-up, reporting satisfactory results related to this procedure, but no stratification of survivorship rates or outcomes between patients who had isolated MATs and the ones with concomitant procedures was performed. Contrarily, a recent systematic review showed that limb malalignment has no negative correlation with PROMs, while 2 of 8 analyzed studies reported that patients who underwent an additional corrective osteotomy were more likely to have short-term graft failure.^5^ Strong evidence was found by Wang et al^21^ showing that concomitant osteotomy for malalignment did not affect the survivorship of MAT at a mean follow-up of 6 years. Last, a retrospective study showed that the performance of concomitant osteotomy was associated with failure to achieve clinically significant outcomes after primary MAT at a minimum of 5 years of follow-up.^19^

Although in the present study patients requiring combined procedures had a slightly higher failure rate than patients with an isolated MAT, the survival rate of almost >90% at 5 years should be considered an excellent result, considering the preoperative condition and the limited treatment options for this group of patients.

Furthermore, it is known that varus malalignment is a risk factor for the development of knee osteoarthritis and that meniscal deficiency in an overloaded compartment further accelerates cartilage degeneration, leaving only a few years to those patients before metal resurfacing options.^1,10,23^ Instead, this study suggested that a combined biological and mechanical approach could significantly postpone the need for total knee replacement (TKR) in young patients with multiple knee comorbidities. Moreover, studies investigating the outcomes of TKR in younger populations found overall implant survivorship of 66% at midterm follow-up and a high incidence of extensor mechanism complications and infections, and middle-aged populations present questionable results in terms of TKR survivorship.^2,16,20^ For all these reasons, the combination of meniscal replacement with osteotomy could be considered an alternative treatment to metal resurfacing in relatively young patients affected by multiple knee comorbidities.

This study has several strengths that are important to highlight. It was specifically designed to compare the clinical outcomes, reoperations, and surgical and clinical failures of patients who underwent arthroscopic MAT with or without combined HTO. The only available study evaluating these outcomes was performed using an open procedure and had a limited sample size compared with the present research.^17^ Moreover, the single-center nature of our study, the standardized surgical procedure, and a mean follow-up of 5.4 years are important aspects that should be considered. There are several differences between this study and the ones mentioned previously that focus on MAT + HTO.^13,17,18^ First, the group of patients analyzed in this study underwent the same surgical procedure, which is a lateral closing-wedge HTO associated with an arthroscopic medial MAT, while the other studies evaluated more heterogeneous groups, which included medial tibial osteotomy and distal femoral osteotomy in some cases, and both medial and lateral meniscal transplantation, not always performed via arthroscopy.

The present study also has some limitations to be considered while interpreting the results. First, the design of the study is a retrospective cohort. In view of the fact that axial malalignment represents a contraindication for performing isolated MAT, and considering that an isolated HTO to unload a meniscus-deficient compartment is considered a suboptimal treatment, it would have been unethical to design a randomized controlled trial and perform medial MAT in a group of patients with meniscal deficiency and severe varus knee.^6^ Second, the patients did not receive a systematic imaging evaluation. Therefore, it is impossible to determine if there was a correlation between the 2 treatments and the status of the transplanted meniscus, the meniscal extrusion grade, and osteoarthritis progression. Moreover, data regarding reoperations and clinical scores were obtained via telephone interviews, with a potential risk of introducing a recall bias. Nevertheless, previous literature has shown that the Lysholm score questionnaire administered via telephone provides accurate data similar to that of a face-to-face interview.^11^ Another limitation is related to the different follow-up periods of the 2 treatment groups; however, a previous study showed that there are minimal clinical differences in the clinical outcomes of MAT in a period of <2 years, and additionally, the Kaplan-Meier analysis helped to further mitigate those differences.^8^ Furthermore, no radiological imaging was performed among the 2 groups during the follow-up period. Finally, because of the study protocol, the survivorship analysis from the clinical failures may not be as precise as the one from the surgical failures. It was impossible to identify the exact time point at which the Lysholm score dropped below 65 points; therefore, the survivorship from clinical failures could be slightly overestimated.

A 5-year survival rate >90%, the good clinical results, and the high patient satisfaction reported in the present study support medial MAT + HTO as a safe and effective procedure for treating symptomatic meniscal deficit and varus malalignment. However, those patients should be informed about the potentially slightly higher failure rate compared with patients who require an isolated medial MAT. This phenomenon is probably because patients with varus malalignment had a more damaged medial compartment in comparison with patients with normal alignment. However, given the lack of radiological images at the final follow-up, it is necessary to further investigate with new studies. Despite these limitations, the present study results help sports medicine surgeons to further understand the outcomes of osteotomy in complex clinical scenarios and further confirm its role with MAT as a powerful tool in joint preservation surgery.

## Conclusion

Patients undergoing medial MAT + HTO showed similar clinical results to patients undergoing isolated medial MAT at midterm follow-up, and thus a surgically addressed malalignment does not represent a contraindication for medial MAT. However, the need for a concomitant HTO is associated with a slightly higher failure rate over time.
